# New Mononuclear Cu(II) Complexes and 1D Chains with 4-Amino-4*H*-1,2,4-triazole

**DOI:** 10.3390/ijms141223597

**Published:** 2013-12-03

**Authors:** Marinela M. Dîrtu, Yves Boland, Damien Gillard, Bernard Tinant, Koen Robeyns, Damir A. Safin, Eamonn Devlin, Yiannis Sanakis, Yann Garcia

**Affiliations:** 1 Institute of Condensed Matter and Nanosciences, Université Catholique de Louvain, Molecules, Solids and Reactivity (IMCN/MOST), Place L. Pasteur, 1, Louvain-la-Neuve 1348, Belgium; E-Mails: marinela.dirtu@uclouvain.be (M.M.D.); boland@chim.ucl.ac.be (Y.B.); damien.gillard@hotmail.com (D.G.); bernard.tinant@uclouvain.be (B.T.); koen.robeyns@uclouvain.be (K.R.); damir.safin@uclouvain.be (D.A.S.); 2 Institute for Advanced Materials, Physicochemical Processes, Nanotechnology & Microsystems, NCSR Demokritos, Athens 15310, Greece; E-Mails: edevlin@ims.demokritos.gr (E.D.); sanakis@ims.demokritos.gr (Y.S.)

**Keywords:** coordination polymers, copper, 1,2,4-triazole, magnetic properties, crystal engineering

## Abstract

The crystal structures of two mononuclear Cu(II) NH_2_trz complexes [Cu(NH_2_trz)_4_(H_2_O)](AsF_6_)_2_ (**I**) and [Cu(NH_2_trz)_4_(H_2_O)](PF_6_)_2_ (**II**) as well as two coordination polymers [Cu(μ_2_-NH_2_trz)_2_Cl]Cl·H_2_O (**III**) and [Cu(μ_2_-NH_2_trz)_2_Cl] (SiF_6_)_0.5_·1.5H_2_O (**IV**) are presented. Cationic 1D chains with bridging bis-monodentate μ_2_-coordinated NH_2_trz and bridging μ_2_-coordinated chloride ligands are present in **III** and **IV**. In these coordination polymers, the Cu(II) ions are strongly antiferromagnetically coupled with *J* = −128.4 cm^−1^ for **III** and *J* = −143 cm^−1^ for **IV** (*H* = −*J*∑*S_i_S_i_*_+1_), due to the nature of the bridges between spin centers. Inter-chain interactions present in the crystal structures were taken into consideration, as well as *g* factors, which were determined experimentally, for the quantitative modeling of their magnetic properties.

## 1. Introduction

There is constant interest in 1,2,4-triazoles and their derivatives, which are known as useful drugs thanks to their outstanding biological activities being used as, antifungal agents [1], plant protection fungicides [2], *etc*. On the other hand, 1,2,4-triazoles are also synthesized for other purposes, for instance in coordination chemistry as metal-azolate frameworks [3], or azole coordination polymers [4,5]. A variety of coordination networks can be designed thanks to the various coordination modes of 1,2,4-triazole-containing building blocks [4]. In particular, numerous oligonuclear and polymeric Cu(II) complexes have been reported with 4-amino-4*H*-1,2,4-triazole (NH_2_trz) [6–17], presumably because it contains a primary –NH_2_ group, which might be further used to functionalize the obtained products. In particular, a few examples of 1D chains were proposed as structural models of the corresponding Fe(II) complexes, which are known to present exceptional spin transition behavior, sometimes occurring around room temperature [18–20]. This includes [Cu(μ_2_-NH_2_trz)_3_](BF_4_)_2_·H_2_O [21], [Cu(μ_2_-NH_2_trz)_3_](BF_4_)(SiF_6_)_0.5_·2H_2_O [21], [Cu(μ_2_-NH_2_trz)_3_]SiF_6_·H_2_O [21], [Cu(μ_2_-NH_2_trz)_3_](NO_3_)_2_·H_2_O [18] and [Cu(μ_2_-NH_2_trz)_3_]ZrF_6_·2H_2_O [22], which all exhibit a polymeric structure where Cu(II) atoms are linked in a 1D chain by the bridging bis-monodentate nitrogen atoms of the 1,2,4-triazole cycles. Interestingly, only two mononuclear Cu(II) NH_2_trz complexes [23,24] are known. Following our continuing interest on the magnetic properties of these systems, we report on the synthesis and crystal structures of two new mononuclear Cu(II) complexes [Cu(NH_2_trz)_4_(H_2_O)](AsF_6_)_2 (_**I**) and [Cu(NH_2_trz)_4_(H_2_O)](PF_6_)_2_ (**II**). We also present the crystal structures and magnetic properties of two 1D chains [Cu(μ_2_-NH_2_trz)_2_Cl]Cl·H_2_O (**III**) and [Cu(μ_2_-NH_2_trz)_2_Cl](SiF_6_)_0.5_·1.5H_2_O (**IV**) whose magnetic properties were studied quantitatively. We also detail the synthesis of a new bis-triazole molecule: *N*,*N*′-bis-(1,2,4-triazole-4-yl)formamidine hydrochloride (**L·HCl**) and describe its crystal structure.

## 2. Results and Discussion

### 2.1. Synthesis

**Scheme 1.**
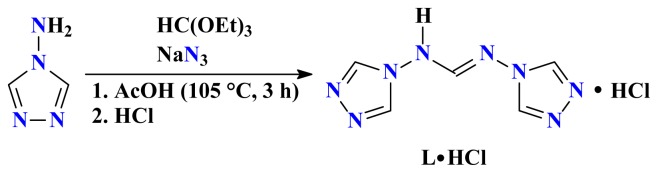
Synthesis of *N*,*N*′-bis-(1,2,4-triazole-4-yl)formamidine hydrochloride.

Two synthetic pathways were followed. Path A is classic with the reaction of [Cu(H_2_O)_6_](AsF_6_)_2_ or a mixture of Cu(CH_3_COO)_2_ H_2_O and (NH_4_)_2_CuCl_4_ 2H_2_O, with NH_2_trz in aqueous MeOH, leading to complexes **I** and **III**, respectively. Path B is more original. It requires first the synthesis of the formamidine **L·HCl** which was obtained by reacting NH_2_trz with NaN_3_ and HC(OEt)_3_ in AcOH in an attempt to get the corresponding triazole-tetrazole molecule. The elemental analysis data suggest the resulting product to be a hydrochloride salt of a novel molecule (Scheme 1). Its ^1^H NMR spectrum recorded in DMSO-*d*_6_ contains two singlet signals at 8.65 and 8.83 ppm, corresponding to the formamidine and triazole CH protons, respectively.

During our attempts to react Cu(II) salts with **L·HCl**, not surprisingly we noticed that this molecule was hydrolyzed in aqueous solution, and proves to be effective to slowly release NH_2_trz and obtain crystals of Cu(II) complexes **II**–**IV**. It should be noted that the formulation of complex **IV** is rather unusual. In addition to the **L·HCl** hydrolysis, SiF_6_^2−^ anions were identified. These anions area actually generated *in situ* by the reaction of BF_4_^−^ anions (provided by the Cu(II) salt) with the glass of the H-tube used to grow crystals of the complex [21]. Furthermore, since **L·HCl** was introduced as a hydrochloride salt, complex **IV** contains Cl^−^ ions, which were provided by the ligand. This might be also true for **III** although CuCl_2_ 2H_2_O was used as a source of Cu(II) ions.

### 2.2. IR and Diffuse Reflectance Spectroscopy

IR spectra of **I**–**IV** each contain a band at about 1630 cm^−1^, corresponding to the amide band of the NH_2_ group (Figure S1). The spectra of **I** and **II** also exhibit an absorption band for the CuOH fragment at around 1000 cm^−1^. There are also bands for the CH, NH_2_ and H_2_O groups in the range from 3000 to 3700 cm^−1^. Additionally, the IR spectra of **I**, **II** and **IV** contain intense broad bands at about 740–800 cm^−1^, arising from AsF_6_^−^, PF_6_^−^ and SiF_6_^2−^ anions, respectively (Figure S1). Diffuse reflectance -spectra of **I**–**IV** exhibit two broad bands in the range 400–900 nm corresponding to d-d transitions (Figure S2). A band from 200 to 400 nm has been attributed to the intraligand NH_2_trz transitions. It should be noted that the IR and diffuse reflectance spectra of **I** and **II** are almost identical (Figures S1 and S2), testifying to isomorphous structures of these two complexes, as was also concluded from Xray diffraction (see next Section).

### 2.3. Structural Aspects

**Figure 1.**
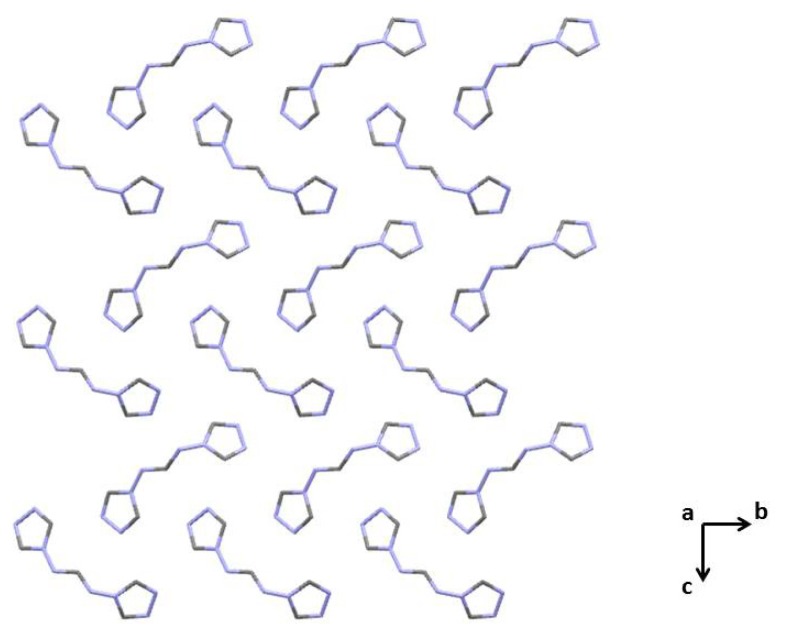
Thermal ellipsoid (50%) plot of L along the *a*-axis. The nitrogen and carbon atoms are depicted in blue and grey, respectively. H-atoms were omitted for clarity.

Recrystallization of **L·HCl** from an aqueous acetone solution leads to the formation of X-ray suitable crystals of **L**, which structure was initially solved and refined in the triclinic space group *P*1, and finally transformed to the monoclinic space group Cc. The molecular structure of **L** is shown in Figure 1. Table S1 in Supplementary Information contains the bond and angle values for **L**. The structure of **L** is stabilized by an intermolecular N–H···N hydrogen bonds between the NH hydrogen atom of the formamidine fragment and the triazole nitrogen atom of a neighboring molecule (Table S2 in Supplementary Information). As a result, the formation of 1D polymeric chains is observed in the structure of **L**.

**Figure 2.**
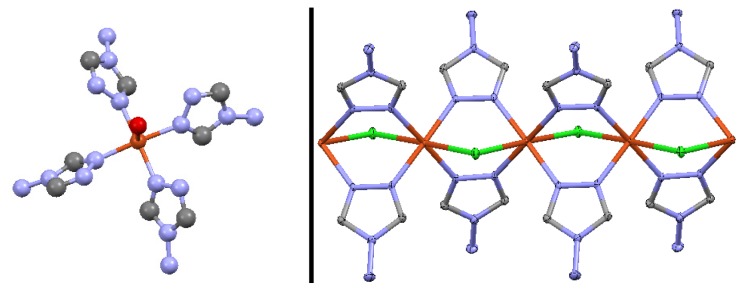
Thermal ellipsoid (50%) plot of **II** (**left**) and **III** (**right**). H-atoms and PF_6_^−^ anions in **II**, and non-coordinated Cl^−^ anions and water molecules in **III** were omitted for clarity. The nitrogen atoms are depicted in blue, the carbon atoms in grey, the chloride ions in green and the copper atoms in red.

Complexes **I**–**IV** were obtained as X-ray suitable blue crystals by slow evaporation of a solvent from their solutions. It should be noted that several crystals of each complex have been checked by a single crystal X-ray analysis, indicating their full identity. Both complexes **I** and **II** crystallize in the tetragonal space group *I*4_1_/a and are isomorphous. Therefore, only the crystal structure of complex **II** will be described. Complexes **III** and **IV** crystallize in the monoclinic space group *P*2_1_/a and triclinic space group *P*–1, respectively, and each exhibit 1D cationic Cu(II) containing polymeric chains. The molecular structures of **II** and **III** are shown in Figure 2.

The discrete mononuclear complex **II** exhibits a distorted square pyramidal coordination polyhedron (Figure 2). In the basal plane, the Cu(II) atom is coordinated by four nitrogen atoms arising from four different NH_2_trz ligands, while the apical position is occupied by a water oxygen atom (Figure 2). The Cu(II) atom in **II** is almost in the basal plane of the square pyramid and only slightly deviated above the plane (0.1890(4) Å). This is a surprising feature as in square pyramidal complexes the metal atom is usually located significantly above the basal plane [25]. This feature can be explained by the Cu···F interaction between the metal center and one of the fluorine atoms of the PF_6_^−^ anion noted in **II**. Indeed, since the fluorine atom is located below the basal plane, it tends to drag the Cu(II) atom towards the bottom of the pyramid. The average Cu–N bond length is about 2.0 Å (Table S3 in Supplementary Information). The Cu–O bond length is 2.263(3) Å. The N–Cu–N bond angles formed by the nitrogen atoms, corresponding to the NH_2_trz ligands coordinated in a *cis*-arrangement are close to 90°, while the N–Cu–N bond angles formed by the nitrogen atoms in *trans*-position are about 170° (Table S3 in Supplementary Information). The N–Cu–O bond angles are about 95° (Table S3 in Supplementary Information).

**Figure 3.**
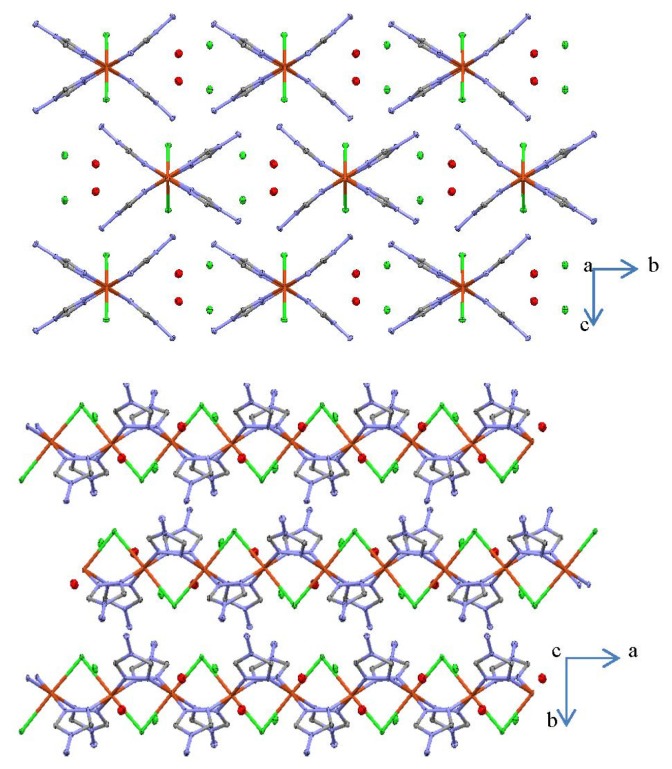
Thermal ellipsoid (50%) plot of **III** along the *a* (**top**) and *c* axes (**bottom**). The nitrogen atoms are depicted in blue, the carbon atoms in grey, the chloride ions in green and the copper atoms in red. H-atoms were omitted for clarity.

Complexes **III** and **IV** each consist of 1D cationic Cu(II)-containing polymeric chains (Figures 2 and 3). The asymmetric unit of **IV** contains two non-equivalent Cu(II) atoms. Cu···Cu separations (~3.60–3.65 Å) in both complexes exceed the sum of van der Waals radii of Cu(II) which indicates the absence of any distinct intramolecular Cu···Cu interactions. Each metal center in **III** and **IV** is coordinated by two chlorine atoms in *trans*-configuration and by four nitrogen atoms arising from four different NH_2_trz ligands (Figure 3). As a result, the formation of the distorted coordination octahedron is observed for both polymeric complexes. It should be highlighted that the structures of **III** and **IV** are unusual, with only one bridging μ_2_-coordinated chloride ligand linking two neighboring Cu(II) atoms in a polymeric chain, because incorporation of chlorine into the coordination sphere of a transition metal in the triazole chains is known to result in triple bridges containing two μ_2_-coordinated chloride ligands [4]. The latter situation is encountered in the crystal structure of [Cu(Htrz)Cl_2_] (Htrz = 4*H*-1,2,4-triazole) [26], which was the first one for a triazole complex elucidated by single crystal X-ray analysis, and in the related chain of [Cu(μ_2_-NH_2_trz)(μ_2_-Cl)_2_] [10]. Thus, depending on the synthesis conditions, it is possible to obtain one or two μ_2_-coordinated chloride ligands in the triple bridge, giving rise to charged or neutral chains, respectively.

**Figure 4.**
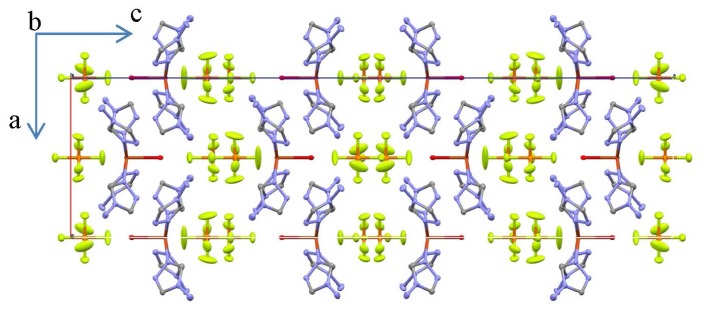
Thermal ellipsoid (50%) plot of **II** along the *b* axis. The nitrogen atoms are depicted in blue, the carbon atoms in grey, the fluoride atoms in green, copper atoms in red and the phosphorus atoms in orange. H-atoms were omitted for clarity.

The Cu–N bond lengths in **III** and **IV** are similar to those found in the crystal structures of **I** and **II**, being about 2.0 Å (Tables S4 and S5 in Supplementary Information). The Cu–Cl bond lengths in **III** are about 2.69–2.78 Å (Table S4 in Supplementary Information), while the same bond lengths in **IV** are slightly shortened, about 2.65–2.72 Å (Table S5 in Supplementary Information). Thus, the equatorial positions on the octahedron are occupied by nitrogen atoms, while the chlorine atoms are in apical positions. Each coordinated Cl^−^ anion exhibits a bridging μ_2_-coordination mode, while NH_2_trz ligands exhibit a bridging bis-monodentate μ_2_-*N*_1_,*N*_2_-coordination mode, linking two neighboring Cu(II) atoms (Figure 2). The N–Cu–N bond angles in each coordination octahedron of **III** and **IV**, formed by nitrogen atoms of the NH_2_trz ligands coordinated in a *cis*-arrangement, are similar to those observed for **I** and **II** and are about 90°. The N–Cu–N bond angles formed by the nitrogen atoms in *trans*-position are significantly enlarged, 178–180° (Tables S4 and S5 in Supplementary Information). The N–Cu–Cl bond angles are in the range 85–95° both in **III** and **IV** (Tables S4 and S5 in Supplementary Information). The Cl–Cu–Cl bond angles are almost straight, 178–180°. The Cu–N–N bond angles defined by the Cu(II) atoms with NH_2_trz ligands are 122–124°, while the Cu–Cl–Cu bond angles are about 84° in both polymeric complexes (Tables S4 and S5 in Supplementary Information). The Cu(II) atoms are located almost in the same plane with the triazole rings. There are two distinguishable octahedron orientations along the polymeric chains resulting in alternate *ababa...* order. Depending on the presence of intrachain ligand interactions, both *ababa...* [18,21,22] and *abcbab...* [27] order may be found in Cu(II) chains with triple μ_2_-*N*_1_,*N*_2_-triazole bridges. In complexes, **III** and **IV**, the lack of direct intrachain ligand···ligand interactions, leads to the more regular *ababa...* order. It should be noted, that the angles between the planes formed by the triazole fragments coordinated to two Cu(II) atoms within a chain in **III** and **IV** are 74.23(13) and 66.55(12)°, respectively. This is considerably higher compared to Cu(II) μ_2_-*N*_1_,*N*_2_-1,2,4-triazole chain complexes (e.g., 50.9(2)° for [Cu(μ_2_-NH_2_trz)_3_]ZrF_6_ H_2_O [21]). The supramolecular aggregation in the structure of **II** consists of head-to-tail double sheets of mononuclear building units (Figure 4). Counter anions are located between double sheets, which are linked by a dense hydrogen bonding network involving amino groups of NH_2_trz and the PF_6_^−^ anions (Table S2 in Supplementary Information). Each amine group is hydrogen bonded to two counter anions and one neighboring amine group. The hydrogen bonding between amine groups links mononuclear complexes of the same sheet (Figure 4). Each counter anion forms N–H···F hydrogen bonds with several mononuclear species, generating links between complexes of the same sheet and complexes corresponding to different double planes. Although the hydrogen atoms of coordinated H_2_O were not located, interatomic distances between oxygen atoms of the water molecules of the sheet and the lone pair of the amine groups, corresponding to a further sheet of the same double plane, indicate the possible existence of hydrogen bonding between these ligands (predicted by the Mercury [28] software). Such hydrogen bonding was proposed by Yi *et al*. [24] to explain the double sheet structure.

**Figure 5.**
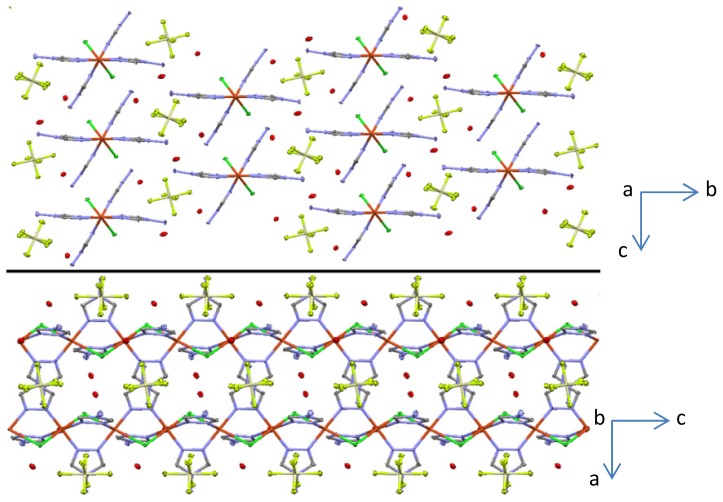
Thermal ellipsoid (50%) plot of **IV** along the *a* (**top**) and *b* (**bottom**) axes. The nitrogen atoms are depicted in blue, the carbon atoms in grey, the fluoride atoms in light green, the chloride ions in green, the copper atoms in red, the silicium atoms in yellow and the oxygen atoms are depicted in red. H-atoms were omitted for clarity.

The crystal packing in **III** and **IV** is made of parallel Cu(II) chains, between which non-coordinated counter anions and water molecules are located (Figures 3 and 5). The Cu···Cu distances in **III** are 3.6532(16) Å, while the same separations in **IV** are insignificantly shortened to 3.5994(16) and 3.6106(16) Å (Table 1), which was expected since no change of the first coordination sphere is noticed between **III** and **IV**. These values are about 0.08–0.14 Å longer than those for the similar Cu(II) chains, containing μ_2_-*O* or μ_2_-*N* bridging atoms, [11–13] but significantly shorter than the Cu···Cu distances in the Cu(II) chains with three μ_2_-1,2,4-triazole derivatives [27,29]. A shorter Cu···Cu distance of 3.40 Å is found when only one μ_2_-*N*_1_,*N*_2_-1,2,4-triazole is bridging as observed for [Cu(Htrz)Cl_2_] [26]. The dihedral angles, formed by the μ_2_-NH_2_trz ligands linking two neighboring Cu(II) atoms, are about 5° and 12° in **III**. The former angle in **IV** is significantly reduced and of 0–2°, while the latter angle is very similar, 12–14°. These values are in a range characteristic for similar Cu(II) chains [12–14]. However, incorporation of the third μ_2_-1,2,4-triazole derivative into the Cu(II) chain leads to a significant increase of the third dihedral angle, which might be explained by steric demands [27,29].

The presence of the amine groups, chloride ions and water molecules favors hydrogen bonding (Table S2 in Supplementary Information). Each chain is linked to a further two chains by the N–H···Cl(coordinated) and N–H···Cl(non-coordinated) ···H–N hydrogen bonding. Moreover, the N–H···Cl···H–O–H···Cl···H–N hydrogen bonds create connections to four neighboring chains. Thus, each chain is hydrogen bonded to six others in the structure of **III**. The crystal packing of **IV** consists of a dense hydrogen bonding network, which links neighboring chains (Table S2 in Supplementary Information). Parallel chains are linked by the multiples N–H···Cl and N–H···N hydrogen bonds. The N–H···O–H···F–Si–F···H–N and N–H···F–Si–F···H–N hydrogen bonds connect the NH_2_trz ligands throughout the whole network. Finally, rows of the SiF_6_^2−^ anions are bound by F···H–O–H···F hydrogen bonding. As a result of dense hydrogen bonding network is observed.

**Figure 6.**
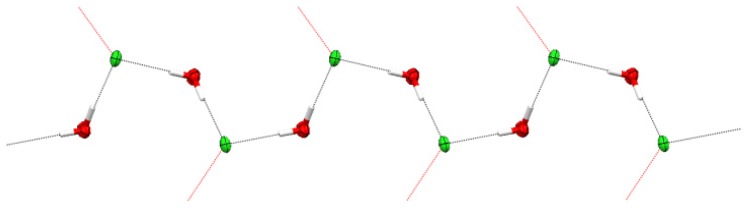
Thermal ellipsoid (50%) plot of the 1D polymeric chain formed by the {[Cl(H_2_O)]^−^}*_n_* cluster in the structure of **III**.

In **III**, voids between metal-containing chains are occupied by water-chloride {[Cl(H_2_O)]^−^}*_n_* supramolecular chains propagated by hydrogen bonds in a meander-like manner (Figure 6, Table S2 in Supplementary Information). These clusters are connected to cationic chains through hydrogen bonds formed between non-coordinated chloride ions and one of the NH_2_ hydrogen atoms of the NH_2_trz ligands (marked by red dash lines in Figure 6, Table S2 in Supplementary Information). Similar structural arrangements of the water-chloride clusters, but in a pure zigzag manner, were observed for Co(II) and Pd(II) complexes [30,31].

### 2.4. Magnetic Properties

**Figure 7.**
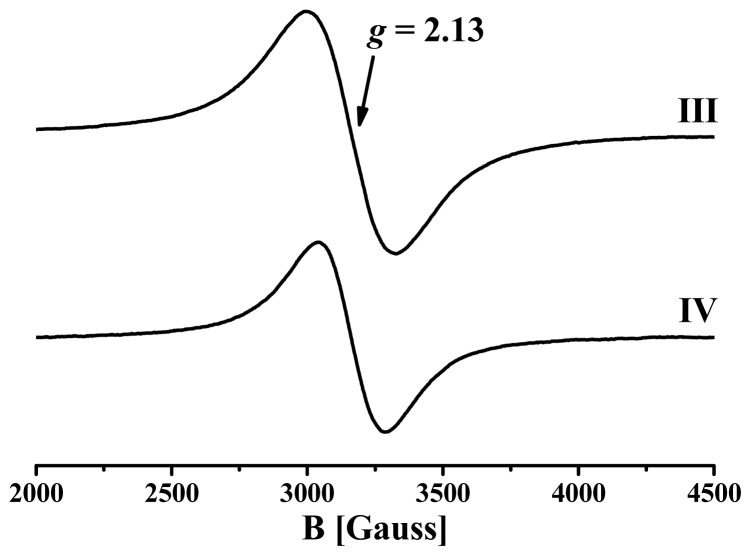
Room temperature X-band EPR spectra from powdered samples of **III** and **IV**.

Since no cooperative magnetism was expected for the mononuclear complexes **I** and **II**, magnetic properties were only investigated for polymeric complexes **III** and **IV**. Electron Paramagnetic Resonance (EPR) spectroscopy at X-band was used to determine the Lande *g*-factors that were thereafter used for a quantitative analysis of the magnetic properties. Room temperature X-band EPR spectra from powdered samples of **III** and **IV** are shown in Figure 7. The spectra consist of broad derivative-like signals with widths of *ΔH_pp_* = 350 and 245 G for **III** and **IV**, respectively. Both complexes are characterized by a *g* value of 2.13.

**Figure 8.**
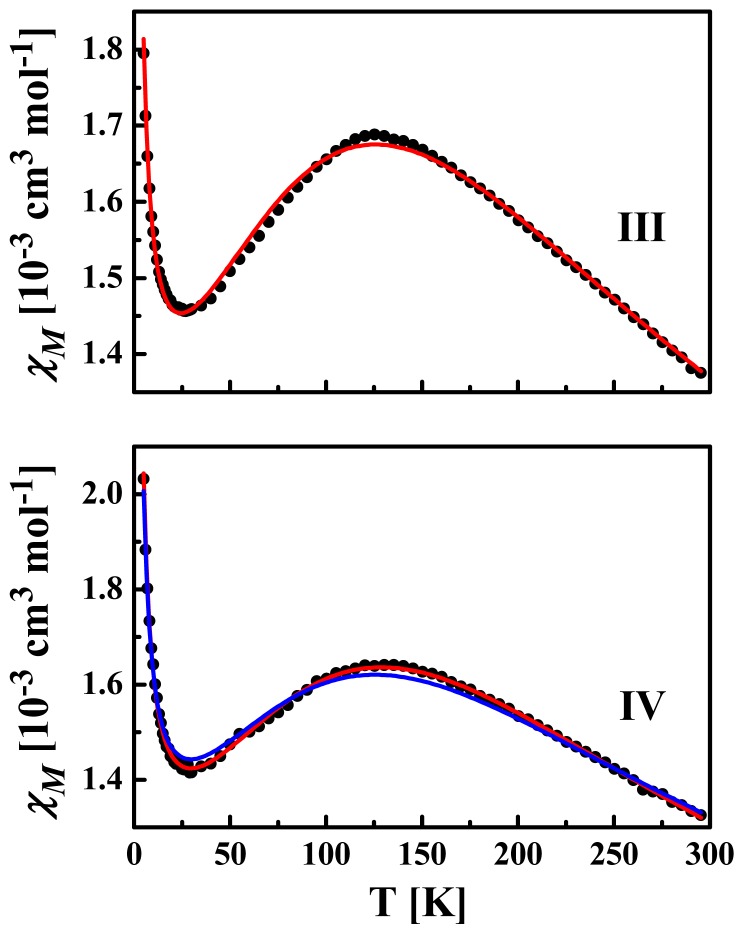
Temperature dependence of molar susceptibility *χ_M_* for powdered samples of **III** and **IV** at 0.1 T. Red solid lines are simulations based on the fitting procedure as discussed in the text. For **IV** the blue line corresponds to the fit obtained assuming *zJ*′ = 0.

Magnetic susceptibility measurements were recorded for powdered samples of **III** and **IV** over the 5–300 K temperature range at an applied field of 0.1 T (Figure 8). At room temperature, the *χ_M_T* values are 0.40 and 0.39 cm^3^ mol^−1^ K for **III** and **IV**, respectively, comparable with that expected for one Cu(II) ion (*S* = 1/2). The temperature profiles of the magnetic susceptibility for both complexes are very similar. As the temperature decreases, *χ_M_* increases smoothly reaching a rather broad maximum at ca. 125 K for **III** and ca. 130 K for **IV**. Below these temperatures, *χ_M_* decreases going through a minimum at ca. 25–30 K before increasing again abruptly (Figure 8).

On the basis of the crystal structures of **III** and **IV**, the magnetic behavior of both complexes was approximated assuming a 1D antiferromagnetic (AF) chain governed by the exchange coupling Hamiltonian: *H* = *–J*∑*S_i_S_i_*_+1_, where *J* is the isotropic interaction parameter. The temperature dependence of the magnetic susceptibility for such a 1D system is adequately described by the Bonner-Fischer approximation [33]:

(1) $$\chi_{M}=\frac{N\beta^{2}g^{2}}{k_{B}T}\left(\frac{0.25+0.074975x+0.075235x^{2}}{1+0.9931x+0.172135x^{2}+0.757825x^{3}}\right)$$

with *x* = |*J*|/*k*_B_*T* and the other terms having their usual meanings.

Since the crystal packing for these 1D chains reveals several inter-chain interactions (Figures 3 and 5), a mean field correction was considered using a corrected susceptibility χ_cor_, where *zJ*′ corresponds to the interchain interaction parameter [34]:

(2) $$\chi_{cor}=\frac{\chi_{M}}{1-\frac{zJ^{\prime }\chi_{M}}{Ng^{2}\beta^{2}}}$$

The increase of χ*_M_* below 30 K is attributed to paramagnetic monomeric impurities and/or short chains after chain breaking [35,36] with *S* = 1/2, for which the magnetic susceptibility, χ_IMP_, follows the Curie law. Overall, the susceptibility data were best fitted following the equation: χ*_M_* = (1 − ρ)χ_cor_ + ρχ_IMP_ + χ_TIP_, where ρ is the fraction of paramagnetic impurities and χ_TIP_ is the temperature independent paramagnetism (TIP). The parameters obtained from these simulations are listed in Table 1, considering two situations for **IV**, with or without intermolecular interactions, the second situation being the less favorable according to fitting results (Figure 8).

Despite the similar nature of the [Cu(μ_2_-NH_2_trz)_2_Cl] polymeric unit in **III** and **IV**, the latter exhibits stronger inter-chain magnetic interactions *zJ*′ (Table 1). The strong AF intra-dimer coupling *J* determined in **III** and **IV** is much higher than those of previously reported 1D Cu(II) complexes made of two μ_2_-*N*_1_,*N*_2_-NH_2_trz and another bridging atom, *i.e.*, N in [Cu(μ_2_-NH_2_trz)_2_(μ_2_-N_3_-N,N)]NO_3_[11], or [Cu(μ_2_-NH_2_trz)_2_(μ_2_-NCS-N)]ClO_4_·0.5H_2_O [12], and O in [Cu(μ_2_-NH_2_trz)_2_(μ_2_-NO_3_)]NO_3_ (Table 1) [13]. These results allow us to evidence an intermediate case between two structural situations by disclosing the crystal structures of the chain compounds **III** and **IV** which show a new bridge type of formula [Cu(NH_2_trz)_2_Cl]^+^. Interestingly, this configuration reveals higher intra-dimer coupling constants compared to the initial case where Cu(II) chains, of formula [Cu(Rtrz)_3_]^2+^, made of three Rtrz bridges show very weak |*J*| values [27,29], and the third case where neutral Cu(II) chains [Cu(NH_2_trz)Cl_2_], show weak *J* values [10,32]. Despite the high *J* values of **III** and **IV**, the record ones reported so far for this substance class are hold by Drabent *et al*. for [Cu(μ_2_-OH)(μ_2_–XPhtrz)]BF_4_·H_2_O and [Cu(μ_2_-OH) (μ_2_-XPhtrz)(H_2_O)]NO_3_ (X = Cl, Br; XPhtrz = *N*-[(*E*)-(4-*R*-methylidene]-4*H*-1,2,4-triazol-4-amine) which range from −391(3) to −608(2) cm^−1^[35]. Ab initio calculations would be useful to appreciate the origin of the magnitude of the coupling constants in these model Cu(II) chain compounds.

## 3. Experimental Section

### 3.1. General Procedures

Infrared spectra (KBr) were recorded with a FTIR-8400S SHIMADZU spectrophotometer (Shimadzu, Kyoto, Japan) in the range 400–3600 cm^−1. 1^H NMR spectra in DMSO-*d*_6_ were obtained on a Bruker Avance 300 MHz spectrometer (Billerica, MA, USA) at 25 °C. Chemical shifts are reported with reference to SiMe_4_. Diffuse reflectance spectra were obtained with a Varian Cary 5E spectrometer (Palo Alto, CA, USA) using polytetrafluoroethylene (PTFE) as a reference. Elemental analyses were performed at University College London, UK. Magnetic susceptibility measurements were performed on a MPMS-5500 Quantum Design instrument (San Diego, CA, USA) in the temperature range 5–300 K. Experimental data were corrected for the sample holder and for the diamagnetic contribution of the sample using Pascal’s constants. X-band EPR spectra at room temperature were recorded on a Bruker ER 200D-SRC X-band spectrometer (Billerica, MA, USA) equipped with an NMR Gaussmeter, and an Anritsu microwave frequency counter. The following conditions were applied: microwave power 59 mW, modulation amplitude 25 Gpp, microwave frequency 9.42 GHz.

### 3.2. Syntheses

***N*,*N*′-bis-(1,2,4-triazole-4-yl)formamidine hydrochloride** (**L·HCl**): A solution of NH_2_trz (4.9 g, 58.3 mmol), HC(OEt)_3_ (9.8 g, 66.1 mmol) and NaN_3_ (3.9 g, 60 mmol) in AcOH (25 mL) was heated at 105 °C for 3 h. The reaction mixture was then cooled and 37% aqueous HCl (5.8 g, 58.3 mmol) was added. The resulting solution was filtered and concentrated under reduced pressure to give a yellowish solid, which was washed by hot EtOH (80 mL) to afford a white solid. Yield: 1.5 g (12%). mp > 200 °C. IR: *ν* = 3105 (*m*), 3095 (*m*), 3068 (*m*), 3025 (*w*), 2971 (*vw*), 1657 (*s*), 1491 (*m*), 1447 (*w*), 1393 (*m*), 1362 (*w*), 1318 (*m*), 1285 (*m*), 1264 (*m*), 1173 (*m*), 1055 (*s*), 990 (*m*), 959 (*m*), 947 (*m*), 899 (*w*), 872 (*m*), 833 (*m*), 764 (*m*), 614 (*s*), 532 (*w*) cm^−1. 1^H NMR: *δ* = 8.65 (s, 1H, CH), 8.83 (s, 4H, CH, 1,2,4-triazole) ppm. Anal. Calc. for C_5_H_7_ClN_8_ (214.62): C 27.98, H 3.29, N 52.21. Found: C 27.63, H 2.99, N 51.11. Crystals of **L** suitable for a single crystal X-ray analysis were obtained by dissolving 0.2 g of **L·HCl** in H_2_O–acetone (5 mL; 50:50, *v*/*v*) solution, which was slowly evaporated at room temperature affording single crystal on standing (4 days).

**[Cu(NH_2_trz)_4_(H_2_O)](AsF_6_)_2_** (**I**): A solution of NH_2_trz (0.252 g, 3 mmol) in MeOH (10 mL) was added drop wise to a solution of [Cu(H_2_O)_6_](AsF_6_)_2_ (0.550 g, 1 mmol) in MeOH (5 mL). The resulting blue precipitate was filtered off, washed with MeOH and dried in a desiccator. A small amount of a solid material (0.050 g) was dissolved in water (100 mL). Crystals suitable for a single crystal X-ray analysis were obtained on standing (12 months) with slow evaporation of the solvent. Yield: 0.376 g (63%). Anal. Calc. for C_8_H_18_As_2_CuF_12_N_16_O (795.71): C 12.08, H 2.28, N 28.16. Found: C 12.17, H 2.23, N 28.24.

**[Cu(NH_2_trz)_4_(H_2_O)](PF_6_)_2_** (**II**): **L·HCl** (0.300 g, 1.39 mmol) was dissolved in water (10 mL). Then a solution of KPF_6_ (0.208 g, 1.13 mmol) and CuCl_2_·2H_2_O (0.097 g, 0.57 mmol) in water (10 mL) was added dropwise leading to a precipitate. To the resulting mixture, additional water (500 mL) was added and the solution was heated until the precipitate completely dissolved. Crystals suitable for a single crystal X-ray analysis were obtained on standing (8 months) with slow evaporation of the solvent and slow delivery of NH_2_trz by hydrolysis of **L**. Yield: 0.285 g (74%). Anal. Calc. for C_8_H_18_CuF_12_N_16_OP_2_ (707.8): C 13.57, H 2.56, N 31.64. Found: C 14.37, H 2.52, N 33.30.

**[Cu(μ_2_-NH_2_trz)_2_Cl]Cl·H_2_O** (**III**): Path A: A solution of Cu(CH_3_COO)_2_·H_2_O (0.106 g, 0.053 mmol) in water (5 mL) was added to a solution of (NH_4_)_2_CuCl_4_·2H_2_O (0.147 g, 0.053 mmol) in the same solvent (5 mL). The resulting blue solution was added to a solution of NH_2_trz (0.134 g, 0.159 mmol) in MeOH (5 mL). Then the mixture was cooled in ice till the blue solid was precipitated. The obtained solid material was filtered off and dissolved in water (50 mL) upon heating. Crystals suitable for a single crystal X-ray analysis were obtained on standing (several weeks) after slow evaporation of the solvent. Yield: 0.114 g. Path B: **L·HCl** (0.300 g, 1.39 mmol) was dissolved in water (10 mL). Then a solution of CuCl_2_·2H_2_O (0.097 g, 0.57 mmol) in water (10 mL) was added dropwise leading to the formation of a precipitate. To the resulting mixture, additional water (450 mL) was added and the solution was heated until the precipitate completely dissolved. Crystals suitable for a single crystal X-ray analysis were obtained on standing (6 months) after slow evaporation of the solvent and slow delivery of NH_2_trz by hydrolysis of **L**. Yield: 0.128 g (75%). Anal. Calc. for C_4_H_10_Cl_2_CuN_8_O (320.63): C 14.98, H 3.14, N 34.95. Found: C 15.11, H 3.19, N 34.82.

**[Cu(μ_2_-NH_2_trz)_2_Cl](SiF_6_)_0.5_·1.5H_2_O** (**IV**): **L·HCl** (0.300 g, 1.39 mmol) was dissolved in water (6 mL) and inserted in the left arm of a H-tube. A 50% wt. aqueous solution of Cu(BF_4_)_2_ (260 μL, 0.55 mmol) was placed in the right arm of a H-tube. Then the tube was carefully filled with water and sealed to prevent evaporation of the solvent. Crystals suitable for a single crystal X-ray analysis were obtained on standing (5 months) after slow delivery of NH_2_trz by hydrolysis of **L** and formation of SiF_6_^2−^ by the reaction of BF_4_^−^ with the glass tube. Yield: 0.137 g (34%). Anal. Calc. for C_8_H_22_Cl_2_Cu_2_F_6_N_16_O_3_Si (730.44): C 13.15, H 3.04, N 30.68. Found: C 13.28, H 2.97, N 30.53.

### 3.3. X-ray Crystallography

The X-ray intensity data were collected with a MAR345 image plate (Norderstedt, Germany) using Mo-K_α_ (λ = 0.71069 Å) radiation. The crystal was chosen, mounted in inert oil and transferred quickly to the cold gas stream for flash cooling. Crystal data and data collection parameters are summarized hereafter. The unit cell parameters were refined using all the collected spots after the integration process. The data were not corrected for absorption (for **L**, **III**, **IV**), but the data collection mode partially takes the absorption phenomena into account. The structures were solved by direct methods with SHELXS [37]. The structure was refined by full-matrix least-squares on F_2_ using SHELXL [37]. All non-hydrogen atoms were refined with anisotropic temperature factors. All hydrogen atoms were placed on calculated positions with temperature factors 1.2 times higher than their parent atoms (1.5 for methyl and OH hydrogens). The Fourier density map for **II** reveals a high residual peak next to the copper coordinated oxygen atom. Inspection of the native Patterson map reveals a large off origin peak (peak height > 50% orgin peak) positioned at 0.5 0 0.25, which is believed to indicate translational disorder. Applying the translation to the Cu atom situated on the 2-fold axis this gives a peak at 0.0000 0.7500 0.0901 or 0.5000 0.2500 0.5901 (+1/2 +1/2 +1/2) (given the **I** centering), which is also the position of the high residual peak in the Fourier map. The crystals (in total three full datasets were measured all giving the same outcome) have all some degree of non-crystallographic translational symmetry and thus consists of two domains which are translated by 1/2 in the *z*-direction. Figures were generated using the Mercury software [14].

**Crystal data for L:** C_5_H_6_N_8_, *M*_r_ = 178.18 g mol^−1^, Monoclinic, space group *C*c, *a* = 3.7400(7), *b* = 21.081(4), *c* = 9.2000(18) Å, β = 100.00(3)°, *V* = 714.3(2) Å^3^, *Z* = 4, *ρ* = 1.657 g cm^−3^, *T* = 293(2) K, *μ*(Mo-Kα) = 0.122 mm^−1^, reflections: 3630 collected, 1345 unique, *R*_int_ = 0.0466, *R*_1_(all) = 0.0399, *wR*_2_(all) = 0.0958.

**Crystal data for II:** C_8_H_16_CuF_12_N_16_OP_2_, *M*_r_ = 705.85 g mol^−1^, tetragonal, space group *I*4_1_/*a*, *a* = *b* = 10.7557(1), *c* = 40.574(1) Å, *V* = 4693.8(1) Å^3^, *Z* = 8, *ρ* = 1.998 g cm^−3^, *T* = 100(2) K, *μ*(Mo-Kα) = 1.204 mm^−1^, reflections: 23,890 collected, 2243 unique, *R*_int_ = 0.046, *R*_1_(all) = 0.0758, *wR*_2_(all) = 0.201.

**Crystal data for III:** C_4_H_10_Cl_2_CuN_8_O, *M*_r_ = 320.65 g mol^−1^, monoclinic, space group *P*2_1_/*a*, *a* = 7.303(3), *b* = 13.838(5), *c* = 11.019(4) Å, *β* = 101.67(2)°, *V* = 1090.6(7) Å^3^, *Z* = 4, *ρ* = 1.953 g cm^−3^, *T* = 101(2) K, *μ*(Mo-Kα) = 2.486 mm^−1^, reflections: 28,160 collected, 2225 unique, *R*_int_ = 0.055, *R*_1_(all) = 0.0316, *wR*_2_(all) = 0.0915.

**Crystal data for IV:** C_8_H_22_Cl_2_Cu_2_F_6_N_16_O_3_Si, *M*_r_ = 730.51 g mol^−1^, triclinic, space group *P*–1, *a* = 7.210(3), *b* = 11.673(4), *c* = 15.271(5) Å, α = 70.35(2), β = 82.16(2), γ = 79.55(2)°, *V* = 1186.4(8) Å^3^, *Z* = 2, *ρ* = 2.045 g cm^−3^, *T* = 105(2) K, *μ*(Mo-Kα) = 2.167 mm^−1^, reflections: 19,683 collected, 4296 unique, *R*_int_ = 0.066, *R*_1_(all) = 0.0504, *wR*_2_(all) = 0.1124.

CCDC 902803 (**L**), 902805 (**II**), 902804 (**III**) and 902806 (**IV**) contain the supplementary crystallographic data. These data can be obtained free of charge via http://www.ccdc.cam.ac.uk/conts/retrieving.html, or from the Cambridge Crystallographic Data Centre, 12 Union Road, Cambridge CB2 1EZ, UK; Fax: (+44) 1223-336-033; or E-Mail: deposit@ccdc.cam.ac.uk.

## 4. Conclusions

In this work, two Cu(II) NH_2_trz mononuclear complexes **I** and **II** have been isolated and structurally characterized, which is rather uncommon for a ligand routinely used in coordination chemistry to afford polynuclear complexes. Our work opens interesting perspectives because such materials are described as potential energy sources for ballistic applications [23,38,39]. We have also presented two novel examples of 1D NH_2_trz chains, **III** and **IV**, where neighboring Cu(II) atoms are linked by two bridging bis-monodentate μ_2_-*N*_1_,*N*_2_-NH_2_trz and one bridging μ_2_-chloride ligand, a configuration which was not yet explored for this family of coordination polymers. Their resulting strong antiferromagnetic coupling has been quantitatively evaluated after considering precise SQUID and EPR measurements.

## Figures and Tables

**Table 1.** Overview of structural characteristics and magnetic data for 1D Cu(II) chains with 1,2,4-triazole ligands.

Complex	*d*(Cu···Cu) (Å)	∠(Cu–N–N–Cu) a (°)	*g*	*J* (cm^−1^)	*P* (%)	*χ*_TIP_ × 10^6^ (cm^3^ mol^−1^)	*zJ*′/*kB* (cm^−1^)	Ref.
[Cu(μ_2_-Htrz)(μ_2_-Cl)_2_]	3.405	−4.67	2.013	−12.4	-	-	-	[26,32]
[Cu(μ_2_-NH_2_trz) (μ_2_-Cl)_2_]	3.4895(5), 3.5210(5)	−5.8(3), 8.2(4)	2.26	−20.4	-	-	-	[10,32]
[Cu(μ_2_-NH_2_trz)_2_Cl] Cl·H_2_O (**III**)	3.653(2)	5.2(3), 11.7(2)	2.13	−128.4	0.65	390	11(5)	This work
[Cu(μ_2_-NH_2_trz)_2_Cl] (SiF_6_)_0.5_ ·1.5H_2_O (**IV**)	3.599(2), 3.611(2)	−1.5(5), −13.9(4); −0.6(5), 11.5(4)	2.13	−143.0	1.02	270	80(4)	This work
			2.13	−139.9	0.90	350	0	
[Cu(μ_2_-NH_2_trz)_2_ (μ_2_-N_3_ -*N*,*N*)]NO_3_	3.5035(8)	−7.8(4), −13.0(4)	2.22(1)	−17.7	-	-	-	[11]
[Cu(μ_2_-NH_2_trz)_2_ (μ_2_-NCS-*N*)] ClO_4_ ·0.5H_2_O	3.470(2)	1.5(6), 2.0(5)	2.21	−51	-	-	-	[12]
[Cu(μ_2_-NH_2_trz)_2_ (μ_2_-NO_3_)]NO_3_	3.5301(5)	−11.4(3), −13.8(3)	2.12	−75.1	-	-	−1.1	[11]
[Cu(μ_2_-hyetrz)_3_] (ClO_4_)_2_ ·3H_2_O b	3.829(2), 3.853(2)	−6.4(7), −10.7(9), −37.5(7); −3.0(7), −11.5(8), −38.0(6)	2.03(1)	−1.18(2)	-	-	-	[27]
[Cu(μ_2_-hyetrz)_3_] (CF_3_SO_3_)_2_ ·H_2_O b	3.8842(4), 3.9354(4)	−3.3(2), −16.3(2), −28.98(19); −11.3(2), −13.8(2), −20.2(2)	2.20(1)	1.45(3)	-	-	-	[29]

a Dihedral angles must be compared by their magnitudes;

b hyetrz = 4-(2′-hydroxyethyl)-1,2,4-triazole.
